# Delayed umbilical cord clamping in rooming-in term newborns: benefits and neonatal outcomes

**DOI:** 10.1590/1984-0462/2026/44/2025378

**Published:** 2026-11-13

**Authors:** Simone Manso de Carvalho Pelícia, Leticia Saori Tutida, Maria Regina Bentlin, João Cesar Lyra, Jose Eduardo Corrente, Ligia Maria Suppo de Souza Rugolo

**Affiliations:** a Universidade Estadual Paulista (UNESP), Faculdade de Medicina de Botucatu, Botucatu, SP, Brazil.

**Keywords:** Rooming-in care, Umbilical cord clamping, Jaundice, Phototherapy, Newborn, Alojamento conjunto, Clampeamento do cordão umbilical, Icterícia, Fototerapia, Recém-nascido

## Abstract

**Objective:**

To evaluate neonatal outcomes associated with delayed umbilical cord clamping (DCC) in term newborns admitted to a rooming-in unit.

**Methods:**

A prospective cohort study was conducted at a university hospital (April–August 2023). Singleton neonates ≥37 weeks’ gestation without congenital malformations or infections were included. Exclusion criteria were intermediate or intensive care admission, umbilical cord clamping time between 30–60 seconds, and hemolytic disease. Participants were divided into two groups based on clamping time: early (0–30 seconds) and delayed (1–3 minutes). Primary outcomes: jaundice requiring phototherapy and length of hospital stay. Secondary outcomes: skin-to-skin contact, breastfeeding within the first hour, hematological parameters, and polycythemia. Data were analyzed using Student’s t-test, χ^2^, and odds ratio (OR) with a 95% confidence interval (CI).

**Results:**

Among the 363 newborns studied (52.14% male; mean gestational age of 39 weeks±1.34 and weight 3240 g±422.54), 281 (77%) underwent DCC. The delayed clamping group showed a significantly lower requirement for neonatal resuscitation (4 vs. 18%), a lower incidence of Apgar scores < 7 at the first minute (0.5 vs. 17%), and a fivefold increase in skin-to-skin contact (IC95% 2.42–12.43). No significant differences were found between groups regarding five-minute Apgar scores, bilirubin levels, the need for phototherapy, weight loss and length of hospital stay. Polycythemia was rare in both groups.

**Conclusions:**

DCC is a beneficial and safe practice for healthy term newborns. It allows for immediate skin-to-skin contact without increasing the risk of jaundice requiring phototherapy or polycythemia, reinforcing its role as a fundamental component of humanized neonatal care.

## RESUMO

**Objetivo:**

Avaliar os desfechos neonatais associados ao clampeamento tardio do cordão umbilical (CTC) em recém-nascidos de termo em alojamento conjunto.

**Métodos:**

Estudo de coorte prospectivo realizado no alojamento conjunto de um hospital universitário entre abril e agosto de 2023. Incluíram-se recém-nascidos ≥ 37 semanas, de gestação única, sem malformações ou infecções congênitas. Excluídos: internação na unidade de cuidados intermediários ou intensivos, clampeamento em 30–60 segundos e doença hemolítica. Conforme o tempo de clampeamento, foram constituídos dois grupos: precoce (0–30 segundos) e tardio (1–3 minutos). Desfechos primários: icterícia com necessidade de fototerapia e tempo de internação. Desfechos secundários: contato pele a pele, amamentação na primeira hora, parâmetros hematológicos e policitemia. Os dados foram analisados pelos testes t de Student, qui-quadrado e razão de chances (OR) com intervalo de confiança de 95% (IC95%).

**Resultados:**

Estudaram-se 363 recém-nascidos (masculino: 52,14%, idade gestacional média de 39 semanas±422,54 e peso 3240 g±1,34); destes, 281 (77%) tiveram CTC. O grupo CTC apresentou menor necessidade de reanimação (4 vs. 18%), menor incidência de Apgar <7 no primeiro minuto (0,5 vs. 17%) e aumento de cinco vezes no contato pele a pele (IC95% 2,42–12,43). Não houve diferenças quanto ao Apgar no quinto minuto, níveis de bilirrubina, necessidade de fototerapia, perda de peso e tempo de internação. A policitemia foi rara nos dois grupos.

**Conclusões:**

O CTC é uma prática benéfica e segura em recém-nascidos de termo saudáveis. Ele permite o contato pele a pele imediato sem aumentar o risco de policitemia ou icterícia, reforçando seu papel fundamental no cuidado neonatal humanizado.

## INTRODUCTION

 The World Health Organization (WHO) recommends delayed umbilical cord clamping (DCC), i.e., 1 to 3 minutes after birth, for full-term and late preterm infants (≥34 weeks) who are vigorous at delivery.^1^ In general, DCC is defined as occurring more than 60 seconds after birth, or once cord pulsations cease. Conversely, cord clamping is considered early when it occurs within the first 30 seconds after birth, and it is termed physiological when performed after the onset of spontaneous respiration. Immediate cord clamping (<15 seconds) is recommended for infants who demonstrate compromised vitality at birth.^2^

 Several studies have demonstrated that DCC promotes a more stable hemodynamic transition after birth, since breathing onset before clamping improves cardiovascular function. Furthermore, placental transfusion to the newborn increases pulmonary blood flow, contributes to blood pressure stabilization, and improves hematological indices in the neonatal period and iron stores in the first months of life.^3-6^

 Despite the benefits of DCC, literature also highlights its potential adverse effects. These effects are largely attributed to the increased red blood cell mass in the newborn, which may raise hematocrit levels and the risk of polycythemia, while subsequent erythrocyte breakdown increases bilirubin production, potentially contributing to jaundice and the need for phototherapy. However, these findings are not consistently reported across studies.^4,7-9^ Although jaundice is a common, often physiological event affecting 50% of full-term and 80% of pre-term newborns,^10^ when serum bilirubin concentrations become significantly elevated, the resulting need for phototherapy can complicate rooming-in care, compromise breastfeeding, and prolong the duration of hospital stay.^11^

 There is a scarcity of national data regarding umbilical cord clamping time and its effects on newborns. One study demonstrated that, even in a baby-friendly hospital that adopts evidence-based practices, the frequency of DCC was low, occurring in only 53.7% of full-term newborns.^12^ Considering the proven benefits of delayed umbilical cord clamping, and the possible undesirable effects related to a higher incidence of jaundice requiring phototherapy treatment and the risk of polycythemia, this study was designed to investigate whether DCC is associated with phototherapy requirement and length of hospital stay among healthy term neonates in rooming-in care. 

## METHOD

 A prospective cohort study was conducted with term infants admitted to the rooming-in unit of a tertiary university hospital from April to August 2023. The study was approved by the institution’s Research Ethics Committee (No. 6.072.081), and the patients’ legal guardians provided written informed consent. 

 All newborns ≥37 weeks of gestational age admitted to the rooming-in unit, from singleton pregnancies, without malformations or congenital infections, were included. 

 Newborns who required admission to the intermediate or intensive care unit, those with umbilical cord clamping performed between 30 and 60 seconds, and those with hemolytic jaundice, evidenced by laboratory tests, were excluded. 

 According to the unit’s protocol, the following laboratory tests are routinely performed on umbilical cord blood at birth: blood typing and direct Coombs test, total bilirubin and fractions, hematocrit, and hemoglobin. All jaundiced newborns underwent serum or transcutaneous bilirubin evaluation; for those requiring phototherapy, levels are reassessed at hospital discharge. 

 Umbilical cord clamping was performed in accordance with current recommendations of the Neonatal Resuscitation Program of the Brazilian Society of Pediatrics;^13^ DCC (1–3 minutes) for newborns ≥34 weeks’ gestational age who were vigorous at birth. Conversely, for non-vigorous infants who did not cry or breathe at birth, or who presented with poor muscle tone, immediate clamping was performed following tactile stimulation by the obstetrician. The timing of umbilical cord clamping was determined by the attending pediatrician and recorded in the neonatal medical records. These infants constituted the early cord clamping (ECC) group, defined as clamping between 0 and 30 seconds. If newborns in this group recovered adequately following initial resuscitation steps, they were placed in skin-to-skin contact, breastfed, and subsequently transferred to the rooming-in unit. Additionally, maternal human immunodeficiency virus (HIV) seropositivity was an indication for ECC. 

 Patients were categorized into two groups based on the timing of umbilical cord clamping: early (≤30 seconds, ECC) and delayed (1–3 minutes, DCC). 

 The following maternal and gestational data were evaluated: age; parity; smoking; gestational diseases (diabetes mellitus, pre-eclampsia); mode of delivery, and time of birth (daytime was defined as 6 a.m. to 6 p.m., and nighttime as 6 p.m. to 6 a.m.). 

 Newborn data analyzed included: gestational age (calculated by the best obstetric estimate: early ultrasound or precise date of last menstruation); sex; birth weight; adequacy of weight for gestational age,^14^ resuscitation at birth (need for positive pressure ventilation); APGAR score <7 at one and five minutes; skin-to-skin contact; breastfeeding in the first hour of life; cord blood tests (total bilirubin and fractions, hematocrit, and hemoglobin); jaundice from the second day of life; bilirubin value in jaundiced newborns (transcutaneous or serum); use of phototherapy; occurrence of polycythemia (serum hematocrit ≥65% or hemoglobin ≥22 g/dL); exclusive breastfeeding; birth weight loss (%), and length of hospital stay. In our unit, phototherapy is indicated according to the 2004 AAP guidelines, and our institutional protocol recommends a follow-up bilirubin assessment 24 hours after treatment initiation.^15^

 The primary outcomes were jaundice requiring phototherapy and length of hospital stay. Secondary outcomes included polycythemia, skin-to-skin contact, breastfeeding in the first hour of life, exclusive breastfeeding and weight loss at discharge. 

 For the sample size calculation, a 3.6% prevalence of the need for phototherapy in term neonates with early and late clamping was considered, according to the meta-analysis by McDonald et al.^4^ Assuming a 2% margin of error and a 95% confidence level (95%CI), a minimum sample size of 331 subjects was required. 

 Descriptive analyses were performed using frequency and association tables. Numerical variables were summarized as mean and standard deviation, whereas categorical variables were expressed as the number and proportion of events. Associations between categorical variables were assessed using the χ^2^ test, while Student’s t-test was used for numerical variables after normality was verified by the Shapiro-Wilk test. For discrete variables, mean comparisons were conducted using a Poisson distribution-based model. The outcomes associated with DCC were evaluated using multiple logistic regression, with the calculation of odds ratios (OR) and their 95%CIs, adjusted for the potential confounders: gestational age and mode of delivery. 

 A 5% significance level or corresponding p-value was applied in every test. All analyses were performed on the SAS for Windows program, v.9.4 (Cary, NC: SAS Institute Inc., 2023). 

 This study was reported in accordance with the Strengthening the Reporting of Observational Studies in Epidemiology (STROBE) statement. 

## RESULTS

 Of the 562 newborns admitted to the rooming-in care unit between April and August 2023, 399 met the inclusion criteria, while 36 were excluded. The final cohort comprised 363 newborns; among these, DCC was performed in 77%, as demonstrated in the study flowchart (Figure 1). During the study period, there were no indications for ECC due to maternal HIV seropositivity. 

**Figure 1:**
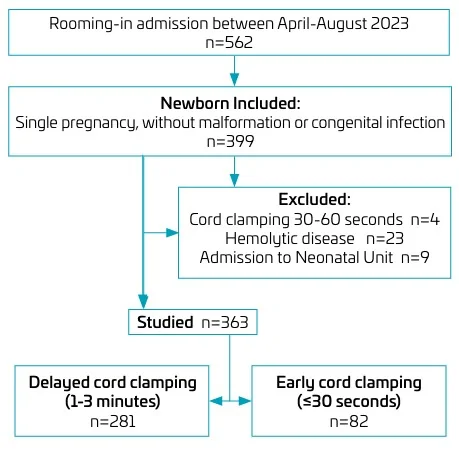
Study flowchart. Source: Prepared by the authors.

 The main maternal and gestational data are shown in Table 1. The only difference between the groups was the presence of meconial-stained amniotic fluid more frequently in ECC. 

**Table 1.** Maternal and gestational data of the early and delayed clamping groups.

Variable	Early (n=82)	Delayed (n=281)	p-value
Maternal age (years) x±SD	26.3 ± 5.9	27.3 ± 6.9	0.194*
Cesarean section (%)	31 (37.8)	142 (50.5)	0.057†
Primigravida (%)	34 (41.5)	94 (33.5)	0.228†
Meconium-stained amniotic fluid (%)	18 (21.9)	22 (7.8)	<0.001†
Pre-eclampsia (%)	12 (14.6)	60 (21.3)	0.236†
Diabetes mellitus (%)	8 (9.7)	32 (11.3)	0.830†
Smoking (%)	9 (10.9)	22 (7.8)	0.501†

*Student’s t-test; †χ^2^.

SD: standard deviation.

Source: Prepared by the authors.

 Mean gestational age was approximately 39 weeks. The incidence of large for gestational age (LGA) and small for gestational age (SGA) infants was 5% and 1.5%, respectively, with no significant difference between groups. Table 2 highlights a higher frequency of resuscitation requirements and 1-minute Apgar scores <7 in the ECC. However, the mean 5-minute Apgar score was 9 in both groups, with no significant differences found, and no infant presented a score below 7. Furthermore, skin-to-skin contact was significantly more frequent in the DCC group (Table 2). 

**Table 2.** Birth data of the early and delayed clamping groups.

Variable	Early (n=82)	Delayed (n=281)	p-value
Gestational age (weeks) x±SD	38.9±1.2	38.8±1.1	0.455*
Birth weight (g) x±SD	3210.9±453.7	3268±414.8	0.292†
Male (%)	48 (58.5)	133 (47.3)	0.097*
Resuscitation at birth (%)	15 (18.2)	11 (3.9)	<0.001*
1-minute Apgar score x±SD	7.5±1.6	8.5±0.8	<0.001*
5-minute Apgar score x±SD	9.0±0.6	9.3±0.5	<0.001*
Apgar <7 at first minute (%)	14 (17.1)	4 (0.5)	<0.001*
Appropriate for gestational age (%)	75 (91.5)	268 (95.1)	0.276*
Breastfeeding 1^st^ hour of life (%)	29 (35.3)	120 (42.5)	0.289*
Skin-to-skin contact (%)	14 (17.1)	107 (37.9)	<0.001*
Total bilirubin cord (mg/dL) x±SD	1.7±0.4	1.8±0.5	0.813†
Hematocrit cord (%) x±SD	49.5±6.9	48.9±5.3	0.465†
Hemoglobin cord (g/dL) x±SD	16.1±2.1	16.2±2.4	0.798†

*Student’s t-test; †χ^2^

SD: standard deviation.

Source: Prepared by the authors.

 The timing of cord clamping was not affected by the time of birth (day or night). DCC was performed in 79.5% of daytime births and in 74% of nighttime births (p=0,287). 

Table 3 shows no significant differences between the groups regarding bilirubin levels and neonatal outcomes. Additionally, only 24 patients had a hospital stay exceeding 72 hours, with similar distribution between groups. 

**Table 3.** Bilirubin levels and neonatal outcomes in the two groups.

Variable	Early (n=82)	Delayed (n=281)	p-value
Total bilirubin during hospitalization (mg/dL) x±SD	8.4±1.7	9.2±1.7	0.062*
Maximum total bilirubin (mg/dL) x±SD	8.9±2.7	9.3±2.1	0.387*
Jaundice (%)	39 (47.5)	149 (52.8)	0.456†
Phototherapy (%)	5 (6)	26 (9)	0.500†
Total bilirubin ≥12 mg/dL (%)	4 (5.0)	16 (7.5)	0.570‡
Total bilirubin at discharge§ (mg/dL) x±SD	8.6±2	9.0±1.8	0.136*
Polycythemia (%)	2 (2.4)	7 (2.5)	0.706‡
Birthweight loss at 48 HOL x±SD (%)	6±3	6.5±2.4	0.131*
Birthweight loss >7% at 48 HOL (%)	28 (34.1)	126 (44.8)	0.110†
Length of hospitalization (days) x±SD	2.1±0.2	2.2±0.1	0.597*
Hospitalization >72 HOL (%)	4 (4.8)	20 (7.1)	0.642‡
Exclusive breastfeeding at discharge (%)	79 (96.3)	263 (93.5)	0.504†

*Student’s t-test; †χ^2^; ‡Fischer; §For neonates who received phototherapy.

SD: standard deviation; HOL: hours of life.

Source: Prepared by the authors.

Table 4 presents the neonatal outcomes associated with DCC, identified through multivariate analysis adjusted for gestational age and type of delivery. DCC was significantly associated with a higher 1-minute Apgar score and a fivefold increase in the odds of skin-to-skin contact. There was no association with other outcomes during hospitalization. 

**Table 4.** Outcomes in the delivery room and during hospitalization associated with delayed umbilical cord clamping: a multivariable logistic regression analysis

Variable		OR*	95%CI	p-value
Delivery room				
	Resuscitation at birth	0.46	0.11–1.96	0.198
	Apgar at first minute	2.47	1.69–3.61	<0.001
	Skin-to-skin contact	5.49	2.42–12.43	<0.001
	Breastfeeding in the first hour of life	1.37	0.68–2.73	0.126
During hospitalization				
	Total bilirrubin ≥12 mg/dL	1.15	0.63–2.11	0.382
	Phototherapy	1.45	0.39–5.44	0.723
	Polycythemia	0.90	0.15–5.42	0.597
	Weight loss >7% at 48 hours	1.08	0.59–1.97	0.523
	Length of hospitalization >72hours	2.43	0.47–12.73	0.401
	Exclusive breastfeeding at discharge	1.63	0.12–3.19	0.270

*Adjusted for gestational age and mode of delivery.

OR: odds ratio; CI: confidence interval.

Source: Prepared by the authors.

## DISCUSSION

 Our results showed that DCC is a frequent (77%), beneficial and safe practice for newborns admitted to rooming-in units. A benefit that warrants highlighting — given the scarcity of reports in the literature — is its positive association with the immediate establishment of skin-to-skin contact at birth. This practice is increasingly encouraged and supported by strong evidence, having been associated with higher rates of exclusive breastfeeding during hospitalization and after discharge, as well as improved cardiorespiratory stability, glycemic control, and thermoregulation.^16^ It should be recognized that during the study period, skin-to-skin contact and breastfeeding in the first hour of life were still incipient in our service; nevertheless, DCC increased the likelihood of skin-to-skin contact by fivefold. 

 A previous study conducted at a baby-friendly university hospital in southern Brazil investigated factors associated with umbilical cord clamping time. Assessing 300 term infants, the study reported that 53.7% underwent DCC (≥60 seconds). Multivariable regression showed that the need for resuscitation was associated with early clamping (OR 1.42; 95%CI 1.16–1.73), while skin-to-skin contact increased the odds of delayed clamping by 24%. The authors suggested that newborns subjected to delayed clamping were in better condition, allowing the team to facilitate skin-to-skin contact in the delivery room.^12^

 Interestingly, the type of delivery did not influence the clamping time, and in almost half of the Cesarean section the umbilical cord was clamped after one minute. The literature raises questions about the ideal time for cord clamping during Cesarean section, particularly in balancing maternal safety with potential benefits for the newborn. Randomized clinical trials and systematic reviews show that delayed cord clamping in Cesarean section does not increase postpartum maternal bleeding and improves hematological indices in newborns, without increasing the need for phototherapy. However, the definition of delayed cord clamping time varies, with studies on the physiology of placental transfusion in Cesarean section showing reverse flow in the umbilical cord after 40 seconds of birth;^17^ thus, many authors consider delayed cord clamping in Cesarean section to be after 30 seconds.^18-21^

 Meconium-stained amniotic fluid was three times more frequent in the ECC than in the DCC group. This finding is likely attributable to the clinical team’s apprehension regarding the possibility of fetal distress and the perceived need for immediate resuscitation. It is noteworthy that the Brazilian Society of Pediatrics’ Neonatal Resuscitation Program mandates that the need for resuscitation be determined by vitality at birth, irrespective of the presence of meconium-stained amniotic fluid.^13^ However, in our study, in addition to the higher frequency of meconium-stained amniotic fluid, the need for resuscitation was four times higher in the ECC group than in the DCC group, which supports the categorization of the former group. 

 As expected, neonatal resuscitation and a one-minute Apgar score <7 were more frequent in the early clamping group, supporting the appropriateness of this practice, since the newborns in this group do not exhibit good vitality at birth. Notably, however, the overall frequency of resuscitation in this group was low, which may be explained by the characteristics of the study population involving only newborns in rooming-in, i.e., those who needed at most the initial steps for cardiorespiratory stabilization. At 5 minutes, no newborn had an Apgar score below 7, and the mean Apgar scores were 9 in both groups, consistent with previous reports in the literature.^4,12,22^

 Although not previously reported in the literature, we hypothesized that the time of birth could influence clinical practice regarding the timing of umbilical cord clamping, potentially reflecting differences between daytime and nighttime delivery room staffs. However, this hypothesis was not confirmed, as no differences in clamping time were observed according to the time of birth, reflecting consistent care provided in a teaching hospital with a permanent, highly trained team. 

 In evaluating the outcomes associated with delayed cord clamping, this practice stands out as both beneficial and safe, consistent with the majority of the literature.^2,16,22-24^ At birth, its benefits include a significantly higher likelihood of improved vitality in the first minute of life and a fivefold increase in the occurrence of skin-to-skin contact. Furthermore, the safety of this practice is underscored by the absence of adverse effects on hematological parameters, the prevalence of jaundice, the need for phototherapy, weight loss, or the length of hospital stay. 

 Cord clamping time did not affect total bilirubin, hematocrit, or hemoglobin levels in umbilical cord blood. The literature consistently shows, for both vaginal and cesarean deliveries, that newborns subjected to DCC present higher hematocrit and hemoglobin levels during the first 72 hours of life.^7,8,19,21,25^ However, these parameters have not been widely reported specifically in cord blood, marking a potential contribution of our study. 

 Although measuring bilirubin in umbilical cord blood is uncommon, it represents an interesting, non-invasive method that may help predict neonatal jaundice. However, the optimal cutoff has not been established, with suggested values ranging from 1.8 to 2.5 mg/dL.^26^

 In our study, cord clamping for 1–3 minutes did not increase neonatal jaundice, the need for phototherapy, or length of stay in the rooming-in unit, contrasting with the study of Yang et al. that found higher bilirubin levels after DCC in full-term newborns,^27^ and a meta-analysis including 2324 term infants that reported significantly fewer infants requiring phototherapy for jaundice with ECC compared to DCC (relative risk — RR 0.62, 95%CI 0.41–0.96).^4^ However, several studies subsequently reported no differences in either bilirubin levels or need of phototherapy, which aligns with our results, reinforcing the safety of this practice.^7,18,19,22^ Notably, jaundice occurred in approximately 50% of rooming-in newborns, consistent with literature reports, with average and peak total bilirubin levels between 8–9 mg/dL on days 2–3. Few newborns had levels ≥12 mg/dL, explaining the low phototherapy rates and the discharge of approximately 95% of newborns within 72 hours. 

 Regarding polycythemia, this was a rare event in our study, not influenced by the cord clamping time. The literature remains inconclusive regarding the risk of polycythemia with DCC.^22,23^ The concern is greater for newborns with fetal growth restriction, as DCC may increase polycythemia, although only a small percentage requires treatment.^28^

 A key limitation of this study is group composition: in the ECC group, it was not possible to determine how many infants underwent immediate clamping, and the delayed group had a wide range of clamping times, which may have influenced the results. However, there was a clear distinction between the early (≤30s) and delayed (1–3 min) groups, and maintaining a range rather than an absolute time point might lead to better adherence to cord clamping protocol. Uncertainty persists regarding the optimal timing of clamping. The variability in timing definitions for early and delayed cord clamping (DCC) is a consistent finding in the literature, which may account for the discrepancies across studies regarding the effects of DCC on hematological parameters, the occurrence of jaundice, and the requirement for phototherapy. Few studies have compared different timings for DCC. A randomized controlled trial comparing cord clamping at 15 seconds, 1 minute, and 3 minutes in term infants revealed that clamping at 1 or 3 minutes resulted in a similar increase in hematocrit values at 6 and 24–48 hours, similar bilirubin levels, and a decreased prevalence of neonatal anemia.^29^ Another randomized controlled trial, which evaluated the effects of DCC at different time intervals (30, 60, and 120 seconds) in late preterm and term neonates in a low-to-middle-income country showed that DCC at 120 seconds led to higher hematocrit, polycythemia, and a longer duration of phototherapy, suggesting that DCC at 30–60 seconds is a safe and effective intervention.^25^

 Another limitation is that this was an observational study subject to residual confounding. Although we attempted to adjust for potential confounders using a multivariable logistic regression model, the observational nature of the study allows us to demonstrate association but not to establish a cause-and-effect relationship. Nevertheless, the results obtained provided a clear answer to the primary research question. 

 Strengths include the prospective design, adequate sample size, and rigorous hematological monitoring. The well-defined separation between early (≤30s) and delayed (1–3 minutes) cord clamping constitutes a significant methodological strength, as it helps mitigate potential classification bias, often referred to as “gray zone” bias, and strengthens the study’s internal validity. Additionally, this research evaluates clinical benefits beyond laboratory parameters, such as increased skin-to-skin contact and improved neonatal vitality. Highlighting these findings is essential to promote the humanization of neonatal care and enhance the quality of assistance in rooming-in units. 

 In conclusion, DCC is a safe and beneficial practice for healthy term infants in rooming-in units. It provides significant clinical advantages, such as allowing immediate skin-to-skin contact, without increasing the risk of jaundice requiring phototherapy, or polycythemia, nor prolonging hospital stay. These findings underscore its role as a fundamental component of humanized neonatal care in both the delivery room and rooming-in settings. 

## Data Availability

The database that originated the article is available with the corresponding author.
